# Wikipedia Usage Estimates Prevalence of Influenza-Like Illness in the United States in Near Real-Time

**DOI:** 10.1371/journal.pcbi.1003581

**Published:** 2014-04-17

**Authors:** David J. McIver, John S. Brownstein

**Affiliations:** Boston Children's Hospital, Harvard Medical School, Boston, Massachusetts, United States of America; Pennsylvania State University, United States of America

## Abstract

Circulating levels of both seasonal and pandemic influenza require constant surveillance to ensure the health and safety of the population. While up-to-date information is critical, traditional surveillance systems can have data availability lags of up to two weeks. We introduce a novel method of estimating, in near-real time, the level of influenza-like illness (ILI) in the United States (US) by monitoring the rate of particular Wikipedia article views on a daily basis. We calculated the number of times certain influenza- or health-related Wikipedia articles were accessed each day between December 2007 and August 2013 and compared these data to official ILI activity levels provided by the Centers for Disease Control and Prevention (CDC). We developed a Poisson model that accurately estimates the level of ILI activity in the American population, up to two weeks ahead of the CDC, with an absolute average difference between the two estimates of just 0.27% over 294 weeks of data. Wikipedia-derived ILI models performed well through both abnormally high media coverage events (such as during the 2009 H1N1 pandemic) as well as unusually severe influenza seasons (such as the 2012–2013 influenza season). Wikipedia usage accurately estimated the week of peak ILI activity 17% more often than Google Flu Trends data and was often more accurate in its measure of ILI intensity. With further study, this method could potentially be implemented for continuous monitoring of ILI activity in the US and to provide support for traditional influenza surveillance tools.

## Author Summary

Although influenza is largely avoidable through vaccination, between 3,000–50,000 deaths occur in the United States each year that are attributed to this disease. The Centers for Disease Control and Prevention continuously monitor the amount of influenza that is present in the American population and compiles this information in weekly reports. However, because it can take a long time to collect and analyze all of this information, the data that is being reported each week is typically between 1–2 weeks old at the time of publishing. For this reason, we are interested in developing new techniques to determine the amount of influenza in the population that are accurate, can return results in real-time, and can be used to supplement traditional monitoring. We have created a method of estimating the amount of influenza-like illness in the American population, at any time of year, by analyzing the amount of Internet traffic seen on certain influenza-related Wikipedia articles. This method is able to accurately estimate the percentage of Americans with influenza-like illness, in real-time, and is robust to influenza seasons that are more severe than normal and to events that promote much media attention, such as the H1N1 pandemic in 2009.

## Introduction

Each year, there are an estimated 250,000–500,000 deaths worldwide that are attributed to seasonal influenza [1], with anywhere between 3,000–50,000 deaths occurring in the United States of America (US) [2]. In the US, the Centers for Disease Control and Prevention (CDC) continuously monitors the level of influenza-like illness (ILI) circulating in the population by gathering information from sentinel programs which include virologic data as well as clinical data, such as physicians who report on the percentage of patients seen who are exhibiting influenza-like illness [2]. While the CDC ILI data is considered to be a useful indicator of influenza activity, its availability has a known lag-time of between 7–14 days, meaning that by the time the data is available, the information is already 1–2 weeks old. To appropriately distribute vaccines, staff, and other healthcare commodities, it is critical to have up-to-date information about the prevalence of ILI in a population.

There have been several attempts at gathering non-traditional, digital information to be used to predict the current or future levels of ILI, and other diseases, in a population [3]–[11]. The most notable of these attempts to date has been Google Flu Trends (GFT), a proprietary system designed by Google, which uses Google search terms that are correlated with ILI activity in the US to make a estimation of the current level of ILI [12]. Google Flu Trends was initially quite successful in its estimation of ILI activity, but was shown to falter in the face of the 2009 H1N1 swine influenza pandemic (pH1N1) due to much-increased levels of media attention surrounding the pandemic [13]. Similarly, GFT greatly over-estimated ILI activity in the 2012–2013 influenza season, again likely due to that fact that it was a more severe influenza season than normally observed and therefore garnered much media attention [14]. In the face of these obstacles, Google has continued to update and re-evaluate its models [15]–[17].

Although GFT has performed well in the past, with the exception of two high ILI activity time periods, new methods of estimating current ILI activity that are less susceptible to error in the face of media coverage should be sought. Additionally, as the global community continues to become increasingly in favor of open-access data and methods [18], new methods of ILI estimation should be freely available for everyone to investigate and improve upon, unlike GFT, which does not share the search terms it uses in its algorithms (though results are public).

To this end, we have created a method of estimating current ILI activity in the US by gathering information on the number of times particular Wikipedia articles have been viewed. Wikipedia is a massive, user-regulated, online encyclopedia. Launched in 2001, Wikipedia harnesses the power of the online community to create, edit, and modify encyclopedia-like articles that are then freely available to the entire world. Currently operating in 232 languages, Wikipedia has ∼30 million articles available, expanding at approximately 17,800 articles per day, with nearly 506 million visitors per month, representing 27 billion total page views since its launch, and has approximately 31,000 active Wikipedia editors (http://stats.wikimedia.org) [19].

With a wealth of detailed information on an almost limitless range of topics, Wikipedia is ideally suited as a platform that could potentially be of use for legitimate scientific investigation in many different areas. Not only is the information held within Wikipedia articles very useful on its own, but statistics and trends surrounding the amount of usage of particular articles, frequency of article edits, region specific statistics, and countless other factors make the Wikipedia environment an area of interest for researchers. It has previously been shown that Wikipedia can be a useful tool to monitor the emergence of breaking news stories, to track what topics are “trending” in the public sphere, and to develop tools for natural language processing [20]–[23]. Furthermore, Wikipedia makes all of this information public and freely available, greatly increasing and expediting any potential research studies that aim to make use of their data.

The purpose of this study was to develop a statistical model to provide near real-time estimates of ILI activity in the US using freely available data gathered from the online encyclopedia, Wikipedia.

## Methods

### Wikipedia Articles of Consideration

In an attempt to use Wikipedia data to estimate ILI activity in the US, we compiled a list of Wikipedia articles that were likely to be related to influenza, influenza-like activity, or to health in general. These articles were selected based on previous knowledge of the subject area, previously published materials, and expert opinion. In addition to articles that were potentially related to ILI activity, several articles were selected to act as markers for general background-level activity of normal usage of Wikipedia. For example, information was gathered on the number of times the Wikipedia main page (www.en.wikipedia.org/wiki/Main_page) was accessed per day, as a measure of normal website traffic. As well, the Wikipedia article for the European Centers for Disease Control was included in models in an attempt to control for non-American article views. Table 1 displays the Wikipedia articles that were considered for inclusion in our models.

**Table 1:** List of Wikipedia articles selected for investigation for inclusion in ILI estimation models.

Avian influenza*	Influenza Virus B*
Centers for Disease Control and Prevention*	Influenza Virus C*
Common Cold*	Influenza Virus Subtype H1N1
Epidemic*	Influenza Virus Subtype H2N2*
European Centers for Disease Control and Prevention	Influenza Virus Subtype H2N9*
Fever*	Influenza Virus Subtype H3N1*
Flu Season*	Influenza Virus Subtype H3N2*
Human Influenza*	Influenza Virus Subtype H5N1*
Influenza	Influenza Virus Subtype H5N2*
Influenza-like Illness*	Oseltamivir*
Influenza Pandemic	Pandemic
Influenza Research*	Swine Influenza
Influenza Treatment*	Tamiflu*
Influenza Vaccine*	Vaccine
Influenza Virus*	Wikipedia Main Page
Influenza Virus A*	1918 Flu Pandemic*

*Only terms with an asterisk were included in the Lasso regression model.

Wikipedia article view information is made freely available by Wikipedia, under a project called Wikimedia Statistics (http://en.wikipedia.org/wiki/Wikipedia:Statistics), and is available as the number of article views per hour, which may include multiple views on the same article by the same user. A freely available, user-written tool was independently developed to more easily access the information that Wikipedia makes available (http://stats.grok.se), which aggregates article view data to the day-level, and this tool was used to gather total daily article view information. Daily Wikipedia article view data was retrospectively collected beginning at the earliest available date, December 10, 2007, through to August 19^th^, 2013, and then aggregated to the week level, with each week beginning on Sunday.

### CDC and GFT Data

The CDC compiles data on the weekly level of ILI activity in the United States by collecting information from sentinel sites across the country where physicians report on the number of patients with influenza-like illness. CDC ILI data is freely available through ILInet, via the online FluView tool (www.cdc.gov/flu/weekly), and downloadable as week-level data. Google Flu Trends data is also freely available through the Google Flu Trends website (http://www.google.org/flutrends) and is provided weekly at the country and state level. GFT data is the result of Google's proprietary algorithm that uses Google search queries to estimate the level of ILI activity in a given region.

### Data Collection

We gathered Wikipedia article view data beginning from the week of December 10^th^, 2007, the earliest records available, until August 19^th^, 2013. Accordingly, retrospective CDC ILI data and GFT data was obtained for the same period as the Wikipedia article view information, although both the CDC and GFT data extends much further back in time. When aggregated to week-level, all data sources accounted for 296 weeks of retrospective information, capturing five full influenza seasons as well as partial 2007–2008 data. Due to a lapse in the Wikipedia database, article view information is not available between July 13^th^ and July 31^st^, 2008, inclusive. Therefore, the total set of data available accounts for 294 weeks.

### Influenza-Like Illness Modeling

Models to estimate ILI activity using Wikipedia article view information were developed using a generalized linear model framework. The outcome variable, age-weighted CDC ILI activity, is a proportion and is therefore appropriately modeled using a Poisson distribution, and so the Poisson family was used in the GLM framework, with a log-link function. In an attempt to adjust for potential over-fitting, models were run using jackknife resampling. Two principle models were created, which include M_f_, a Poisson model that used the full set of collected Wikipedia article page view data, and M_l_, a Poisson model that used Lasso (Least Absolute Shrinkage and Selection Operator) regression analysis. Lasso regression dynamically and automatically selects predictor variables for inclusion or exclusion by penalizing the absolute size of the regression coefficients toward zero, thereby selecting a subset of predictor variables which best describe the outcome data [24], [25]. To investigate the reliability of the models, we used a split-sample analysis on the M_l_ models to compare how well the Lasso selected predictors for a subset of the data (including years 2007, 2008, 2009, and 2010) accounted for the observed data in the remaining subset (years 2011, 2012, and 2013).

Additionally, each of these aforementioned models were also run while excluding data at key time periods which reflect higher than normal ILI activity or Wikipedia article view traffic (during the early weeks of the 2009 pandemic H1N1 swine influenza pandemic and the unusually severe influenza season of 2012–2013) as a means of investigating the models' ability to deal with large data spikes. By comparing the models with or without higher than normal Wikipedia usage, we can investigate what impact, if any, spikes in Wikipedia activity (potentially caused by increased media reporting of influenza-related events) have on the accuracy of the models, and whether or not these spikes in traffic need to be accounted for. In addition to a factor variable representing the year being included in the models, the month was also controlled for in an effort to adjust for the seasonal patterns that influenza outbreaks exhibit in the United States. All models were investigated for appropriate fit using the Pregibon's goodness-of-link test [26] and by examining Anscombe and deviance residuals. Models were compared to one another by comparing Akaike's Information Criteria, response statistics, and by performing likelihood-ratio tests on the maximum-likelihood values of each model. Goodness-of-fit (GOF) tests, both Pearson and deviance, were tested for; all presented models had GOFs≫0.05. All statistics and models were performed using Stata 12 (Statacorp., College Station, Texas, US).

## Results

Across the 294 weeks of data available, the number of views of each Wikipedia article under consideration showed large variability. As an example of this variation, the mean number of daily views of the “Influenza” article was 30,823, but the total number of views ranged from 3,001–334,016 per day. Some of the articles under investigation had relatively few views, such as “influenza-like illness” with a mean of 1,061 article views per day (range: 0–15,629 views per day), while others had very high numbers of views per day, such as the Wikipedia Main Page, which had a mean of 44 million views per day (range: 7–139 million views per day).

Herein, we will discuss the characteristics of several models in an attempt to use Wikipedia article view information to estimate nationwide ILI activity based on CDC data. We consider a full model (M_f_) that includes all dependent variables that were investigated and a Lasso-selected model (M_l_) that includes only dependent variables chosen as significant by the Lasso regression method.

### Full-Data Models

The M_f_ model, containing all 35 predictor variables (including year, month, CDC page views, ECDC page views, and Wikipedia Main Page views) and 294 weeks of data, resulted in a Poisson model with an AIC value of 2.795. Deviance residuals for this model ranged from −0.971–1.062 (mean: −0.006) and were approximately normally distributed. Although many of the dependent variables showed spikes in page view activity around the beginning of the 2009 pH1N1 event, the M_f_ model was able to accurately estimate the rate of ILI activity, with a mean response value (difference between observed and estimated ILI values) of 0.48% in 2009 between weeks 17–20, inclusive. Overall, the absolute response values for the M_f_ model ranged from 0.00–2.38% (mean: 0.27%, median: 0.16%). In comparison, the absolute response values between CDC ILI data and GFT data ranged from 0.00–6.04% (mean: 0.42%, median: 0.21%). The Pearson correlation coefficient between the CDC ILI values and the estimated values from the M_f_ model was 0.946 (p<0.001). The actual observed range of ILI activity throughout the entire period for which data is available, as reported by the CDC, was from 0.47–7.72%, with a median value of 1.40%. In comparison, the M_f_ model estimated ILI activity for the same period ranged from 0.44–8.37%, with a median value of 1.50%, and the GFT ILI data ranged from 0.60–10.56%, with a median value of 1.72%.

The M_l_ model, which contained 26 variables (including year, month, and CDC page views) that were chosen as significant by the Lasso regression method, resulted in a model with an AIC of 2.764. Deviance residuals for this model ranged from −0.790 to 1.205 (mean: −0.007) and were approximately normally distributed, though less so than in M_f_. The absolute response values for this M_l_ model ranged from 0.00–2.53% (mean: 0.29%, median: 0.18%). During weeks 17–20 of the 2009 pH1N1 event, the mean response value for this model was 0.45%, suggesting it was slightly less accurate over this unusually high article view activity time period than the M_f_ model for the same period. The Pearson correlation coefficient between CDC ILI data and the estimated mean value for the Ml model was 0.938 (p<0.001), and the range of estimated ILI values for this model was from 0.55–8.66%, with a median value of 1.48%.

Split-sample analysis was used to investigate the reliability of the M_l_ model. A Lasso regression model that was trained on data from years 2007–2010, inclusive, and the selected predictor variables were used to estimate the ILI activity for each week in the remainder of the dataset (years 2011–2013, inclusive). The cross-validation Pearson correlation between the actual observed CDC ILI data and the ILI estimates provided by the M_l_ model based on the first subset of data was 0.9854 (p<0.001).

**Figure 1:**
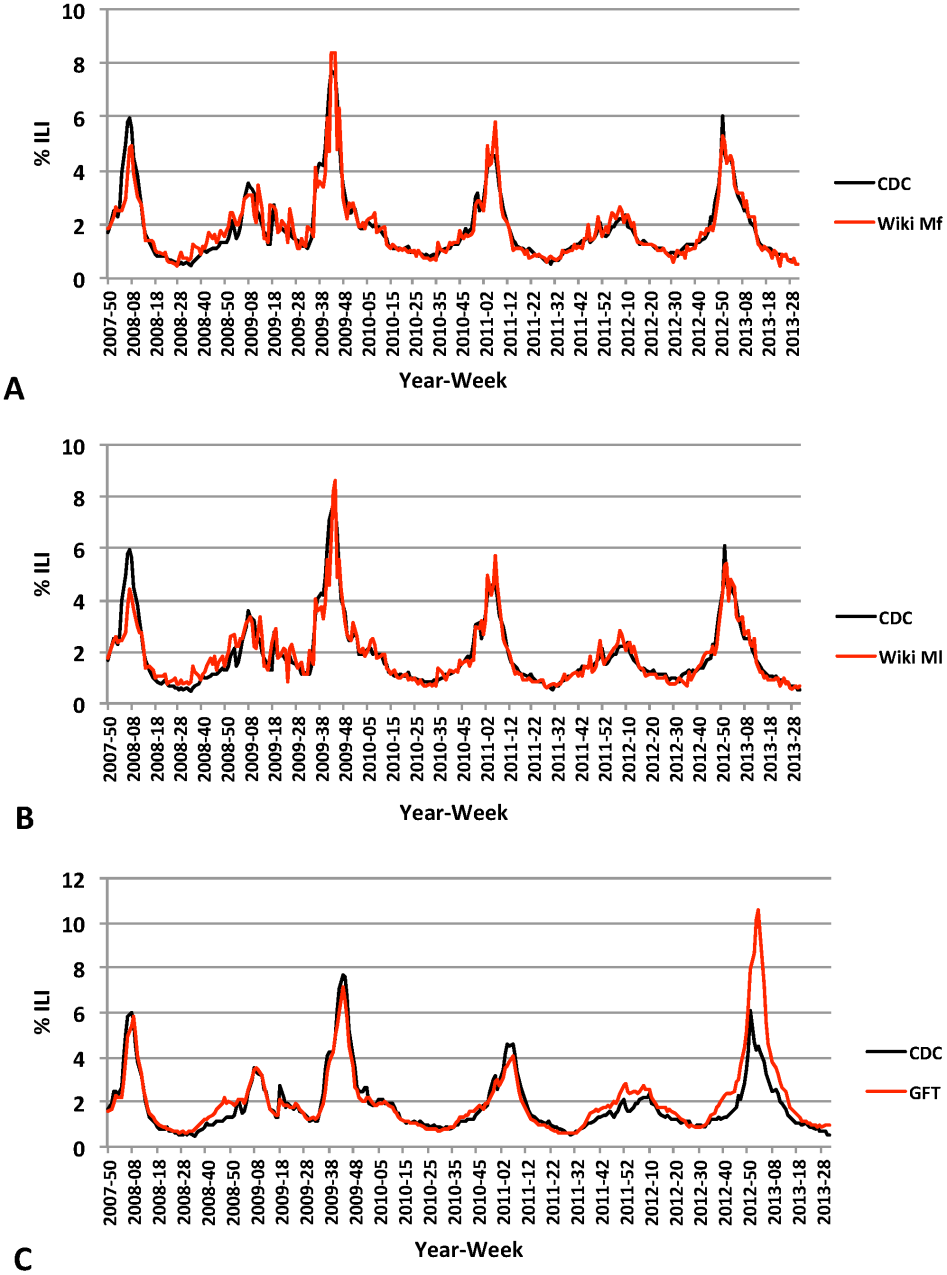
Time series plot of CDC ILI data versus estimated ILI data. (A) Wikipedia Full Model (Mf) accurately estimated 3 out of 6 ILI activity peaks and had a mean absolute difference of 0.27% compared to CDC ILI data. (B) Wikipedia Lasso Model (Ml) accurately estimated 2 out of 6 ILI activity peaks and had a mean absolute difference of 0.29% compared to CDC ILI data,. (C) Google Flue Trends (GFT) model accurately estimated 2 of 6 ILI activity peaks and had a mean absolute difference of 0.42% compared to CDC ILI data.

Figure 1 shows the time series for CDC ILI data, GFT data, and the estimated ILI values from both the M_f_ and M_l_ models.

### Models without Peak Activity

In the following models, data from the beginning weeks of the 2009 pH1N1 event (weeks 17–20, inclusive), which showed large spikes in Wikipedia article views due to increased media attention, were excluded from analyses. As well, because of the higher-than-normal influenza activity of the 2012–2013 influenza season, that data was also removed from analyses, beginning from week 40 of 2012 to week 13 of 2013, inclusive. By running the Poisson models without these high volume time-sections, comparisons can be made to the full models in order to investigate the estimating ability of models in the face of higher-than-normal levels of influenza activity or Wikipedia article views.

When removing the above-mentioned data, the M_f_ model produced an AIC value of 2.772, only marginally smaller than that of the complete M_f_ model, and was comprised of 263 weeks of data. The range of deviance residuals from this model, −0.650 to 0.891, is slightly narrower than the complete M_f_ model, suggesting a better fit. For the truncated Lasso model, the Poisson regression model was refit to only include the available data, and therefore produced a different set of 24 predictor variables. From this model, an AIC value of 2.727 was obtained, with a range of deviance residuals from −0.677 to 1.081, a marginal narrowing over the original M_l_ model. Pearson correlation coefficient values between CDC ILI data and estimated values by the M_f_ and M_l_ models, for peak-truncated data, were 0.958 (p<0.001) and 0.942 (p<0.001), respectively.

### Peak Influenza-Like Illness Estimation

In the United States, seasonal influenza activity usually peaks during January or February. Using the maximum value of the CDC ILI data in a single influenza season as the true peak time and value, we compared the peak value and week for influenza activity as estimated by our two models, M_f_ and M_l_, as well as the Google Flu Trends data. Results are summarized by model and by year in Table 2.

**Table 2:** Comparisons of CDC, M_f_, M_l_, and GFT peak ILI values.

Influenza Season	Year	Week	ILI Value	Referent CDC ILI Value*	% Difference from CDC ILI Value	Peak Agrees with CDC
**2007–2008**						
**CDC Peak**	2008	7	5.98			
**M_f_ Peak**	2008	8	4.94	5.62	0.68	N
**M_l_ Peak**	2008	7	4.43	5.98	−1.55	Y
**GFT Peak**	2008	8	5.81	5.62	0.19	N
**2008–2009**						
**CDC Peak**	2009	7	3.57			
**M_f_ Peak**	2009	12	3.48	2.43	−1.05	N
**M_l_ Peak**	2009	12	3.33	2.43	0.90	N
**GFT Peak**	2009	8	3.50	3.37	0.13	N
**2009–2010**						
**CDC Peak**	2009	43	7.72			
**M_f_ Peak**	2009	43	8.36	7.72	−0.64	Y
**M_l_ Peak**	2009	44	8.66	7.55	1.11	N
**GFT Peak**	2009	43	7.11	7.72	−0.61	Y
**2010–2011**						
**CDC Peak**	2011	4	4.55			
**CDC Peak**	2011	6	4.55			
**M_f_ Peak**	2011	6	5.84	4.55	−1.29	Y
**M_l_ Peak**	2011	6	5.73	4.55	1.18	Y
**GFT Peak**	2011	6	4.08	4.55	−0.47	Y
**2011–2012**						
**CDC Peak**	2012	10	2.39			
**M_f_ Peak**	2012	7	2.68	2.24	−0.44	N
**M_l_ Peak**	2012	7	2.85	2.24	−1.55	N
**GFT Peak**	2011	52	2.86	1.74	1.12	N
**2012–2013**						
**CDC Peak**	2012	51	6.07			
**M_f_ Peak**	2012	51	5.31	6.07	0.76	Y
**M_l_ Peak**	2012	52	5.40	4.65	−1.55	N
**GFT Peak**	2013	2	10.56	4.52	6.04	N

ILI: Influenza-like illness, CDC: Centers for Disease Control and Prevention.

M_f_: Full model, M_l_: Lasso model, GFT: Google Flu Trends.

*Referent values are CDC ILI values for the corresponding week of the estimated ILI peak for M_f_, M_l_, and GFT.

The M_f_ model was able to accurately estimate the ILI activity peak in 3 of 6 influenza seasons for which data is available (2009–2010, 2010–2011 and 2012–2013 seasons), and was within one week of an accurate estimation in another season (2007–2008). The M_l_ model accurately estimated the ILI peak activity week in 2 of 6 seasons (2007–2008 and 2010–2011), and estimated 2 others within a week (2009–2010 and 2012–2013). In comparison, Google Flu Trends data was able to accurately estimate peaks of seasonal ILI activity in 2 of 6 influenza seasons (2009–2010 and 2010–2011 season), and was accurate within one week in 2 other influenza season (2007–2008 and 2008–2009). It should be noted that in the 2010–2011 season, the CDC data peaked at the same ILI percentage at both week 4 and week 6 in 2011, and week 6 was taken to be the true peak, as it agreed with both Wikipedia models and the GFT data. In the 2011–2012 season, the M_f_ and M_l_ models were 3 weeks early in their estimation of peak ILI activity and the GFT data was 10 weeks early. Finally, in the 2012–2013 influenza season, the GFT model was 3 weeks late and grossly over-estimated the severity by greater than 2.3-times.

## Discussion

Weekly ILI values based on Wikipedia article view counts were able to estimate US ILI activity within a reasonable range of error, with CDC data as the gold standard. While the CDC ILI data is routinely used as a gold standard, and is most often the best available source of ILI information for the country, this data source has potential biases of its own. There are over 2,900 outpatient healthcare providers that are registered participants of the CDC's ILI surveillance program, but in any given week, only approximately 1,800 provide ILI surveillance data [27]. As well, the population size/density of the area served by each outpatient healthcare provider is not uniform across locations and may lead to a skew in reporting. Additionally, increased media coverage of influenza may prompt healthcare providers to submit more samples for analysis or to report more potential ILI cases than they may have otherwise. Several models were fit to estimate ILI activity, including a model containing all 32 health-related Wikipedia articles investigated, a Lasso regression model which selected 24 health-related Wikipedia articles of significance, and each of these models were run without high media-awareness time periods representing the beginning of the H1N1 pandemic in spring of 2009 and the higher-than-normal ILI rates of the 2012–2013 influenza season. These models were compared to official CDC ILI values as well as GFT data.

Comparing the M_f_ and M_l_ models, the AIC value was slightly smaller for the M_l_ model, as was its range of estimation residuals. With a highly non-significant likelihood ratio test between the two models, there is no evidence to suggest that the M_f_ model performs better than the M_l_ model, which may be preferred here. However, since there is no cost or energy associated with collecting additional variable information, the full model may warrant continued use to account for the potential event where more health-related Wikipedia articles become useful in ILI estimation. M_f_ and M_l_ models that did not include data for the 2009 spring pH1N1 season and the 2011–2012 peak season resulted in slightly smaller AIC and residual values compared to their full-data counterparts, but did not show large enough improvements in estimates to suggest that higher than normal Wikipedia page view traffic or ILI activity were major factors in the models' ability to estimate ILI activity. This result exemplifies the Wikipedia model's ability to perform well in the face of increased media attention and higher than normal levels of ILI activity, whereas GFT has been shown on several occasions to be highly susceptible to these types of perturbations.

In comparison to GFT data, there are some areas where the Wikipedia models were superior, but others where they were not. Full Wikipedia models were able to estimate the week of peak activity within a season more often than GFT data. Out of the 6 seasons for which data was available, GFT estimated a value of ILI that was more accurate (regardless of whether or not the peak timing was correct) than the M_f_ or M_l_ models in 4 seasons, while the Wikipedia models were more accurate in the remaining 2. These analyses and comparisons were carried out on GFT data that was retrospectively adjusted by Google after large discrepancies between its estimates and CDC ILI data were found after the 2012–2013 influenza season, which was more severe than normal. Even with this retrospective adjustment in GFT model parameters, the peak value estimated by GFT for the 2012–2013 is more than 2.3-times exaggerated (6.04%) compared to CDC data, and was also estimated to be 4 weeks later than it actually was. For this same period, the M_f_ model was able to accurately estimate the timing of the peak, and its estimation was within 0.76% compared to the CDC data.

This study is unique in that it is the first scientific investigation, to the authors' knowledge, into the harnessing of Wikipedia usage data over time to estimate the burden of disease in a population. While Google keeps GFT model parameters confidential, the Wikipedia article utilization data in these analyses are freely available and are open to be modified and improved upon by anyone. Although it has not been investigated here, there is potential for this method to be altered for the monitoring of other health-related issues such as heart disease, diabetes, sexually transmitted infections, and others. While the above mentioned conditions do not have the same time-varying component as influenza, overall burden of disease may potentially be estimated based on the number of people visiting Wikipedia articles of interest. This is an open method that can be further developed by researchers to investigate the relationship between Wikipedia article views and many factors of interest to public health.

Data regarding Wikipedia page views is updated and available each hour, though data in this study has been aggregated to the day level, and then further aggregated to the week level. This was done so that one week of Wikipedia data matched one week of CDC's ILI estimate. In practice, if this Wikipedia based ILI surveillance system were to be implemented on a more permanent basis, it is possible that updates to the Wikipedia-estimated proportion of ILI activity in the United States could be available on a daily or even hourly basis, although this application has not yet been explored. It is hypothesized that hourly updates may have trouble dealing with periods of low viewing activity, such as nighttime and normal sleeping hours, and that the benefit of an hourly update versus a daily update might not be worth the effort involved in its perpetuation. Daily estimates are likely to be of greater use than hourly and hold potential for use as a tool for detecting outbreaks in real-time, by creating an alert when the daily number of Wikipedia article views spikes over a set threshold.

As with any study using non-traditional sources of information to make estimations or predictions, there is always some measure of noise in the gathered information. For instance, the number of Wikipedia article views used in this study represent all instances of article views for the English language Wikipedia website. As such, while the largest proportion of these article views comes from the United States (41%, with the next largest location being the United Kingdom representing 11%), the remaining 59% of views come from other countries where English is used, including Australia, the United Kingdom, Canada, India, etc. Since Wikipedia does not make the location of each article visitor readily available, this makes the relationship between article views and ILI activity in the United States less reliable than if the article view data was from the United States alone. To investigate this bias, it may be of interest to replicate this study using data that is country and language specific. For instance, obtaining Wikipedia article view information for articles that exist only on the Italian language Wikipedia website and comparing that data to specific Italian ILI activity data. Alternatively, the timing and intensities of influenza seasons in English-Wikipedia-using countries apart from the United States could be investigated as potential explanations of model performance. Depending on the timing of influenza activity in other countries, their residents' Wikipedia usage could potentially bolster the presented Wikipedia-based model estimations (if their influenza seasons are similar to that of the United States), or it could negatively impact estimations (if their influenza seasons are not similar to those of the United States). This is an interesting method of comparison and may potentially be explored in future iterations of this method.

If these models continue to estimate real-time ILI activity accurately, there is potential for this method to be used to predict timing and intensity in upcoming weeks. While re-purposing these models could potentially be a significant undertaking, we are interested in pursing this avenue of investigation in future works.

There has been much discussion in popular media recently about the potential future directions of Wikipedia. It has been noted in several papers and reviews that the number of active Wikipedia editors has been slowly decreasing over the past 6 years, from its peak of more than 51,000 is 2007 to approximately 31,000 in the summer of 2013. [19], [28] It has been speculated that the efforts made by the Wikimedia Foundation and it's core group of dedicated volunteers to create a more reliable, trustworthy corpus of information has limited the ability of new editors to edit or create new articles, thereby decreasing the likelihood that a new contributor will become a trusted source of information. Compounding this decrease in active editors, it has become increasingly evident that the vast majority of articles on the English Wikipedia website are both male and Western and European-centric, with comparatively few articles dealing with highly female-oriented topics or other geographic areas. Despite these concerns, the articles relating to influenza that have been investigated in this study are within the scope of the type of Wikipedia articles that are routinely and adequately maintained by long-time editors. The authors hypothesize that any decreases in the number of editors in the Wikimedia domain are unlikely to create significant changes in viewership of the articles of interest for estimating or predicting influenza-like illness, and therefore should not contribute meaningfully to the pursuit of this type of surveillance.

Due to an error in Wikipedia data collection, there were no article view data available between July 13, 2008–July 31, 2008, inclusive, resulting in a time gap of just over 2.5 weeks. Fortunately, this time gap occurred in a traditionally low ILI prevalence time of year, and is not suspected to meaningfully impact analyses.

The application of Wikipedia article view data has been demonstrated to be effective at estimating the level of ILI activity in the US, when compared to CDC data. Wikipedia article view data is available daily (and hourly, if necessary), and can provide a reliable estimate of ILI activity up to 2 weeks in advance of traditional ILI reporting. This study exemplifies how non-traditional data sources may be tapped to provide valuable public health related insights and, with further improvement and validation, could potentially be implemented as an automatic sentinel surveillance system for any number of disease or conditions of interest as a supplement to more traditional surveillance systems.
